# Transduction optimization of AAV vectors for human gene therapy of glaucoma and their reversed cell entry characteristics

**DOI:** 10.1038/s41434-019-0105-4

**Published:** 2019-10-14

**Authors:** Laura Rodriguez-Estevez, Priyadarsini Asokan, Teresa Borrás

**Affiliations:** Department of Ophthalmology, University of North Carolina School of Medicine, Chapel Hill, NC USA

**Keywords:** Molecular biology, Diseases

## Abstract

The trabecular meshwork (TM) of the eye is responsible for maintaining physiological intraocular pressure (IOP). Dysfunction of this tissue results in elevated IOP, subsequent optic nerve damage and glaucoma, the world’s leading cause of irreversible blindness. IOP regulation by delivering candidate TM genes would offer an enormous clinical advantage to the current daily-drops/surgery treatment. Initially, we showed that a double-stranded AAV2 (scAAV2) transduced the human TM very efficiently, while its single-stranded form (ssAAV2) did not. Here, we quantified transduction and entry of single- and double-strand serotypes 1, 2.5, 5, 6, 8, and 9 in primary, single individual-derived human TM cells (HTM). scAAV2 exhibited highest transduction in all individuals, distantly followed by scAAV2.5, scAAV6, and scAAV5. Transduction of scAAV1, scAAV8, and scAAV9 was negligible. None of the ssAAV serotypes transduced, but their cell entries were significantly higher than those of their corresponding scAAV. Tyrosine scAAV2 capsid mutants increased transduction in HTM cultured cells and all TM-outflow layers of perfused postmortem human eyes. These studies provide the first serotype optimization for gene therapy of glaucoma in humans. They further reveal biological differences between the AAV forms in HTM cells, whose understanding could contribute to the development of gene therapy of glaucoma.

## Introduction

Glaucoma has been recently defined as “*a group of ocular disorders with multifactorial etiology united by a clinical characteristic intraocular pressure-associated optic neuropathy”* [1]. In all its forms, glaucoma is the leading cause of irreversible blindness worldwide. A 2010 report from the WHO estimates that 39 million people in the world are blind [2]. The global prevalence of glaucoma in the older population is 4% and by 2040 is estimated to reach 111.8 million people [3]. Although there are cases where a glaucomatous optic neuropathy occurs without elevated intraocular pressure (IOP) [4], it is well-established that elevated IOP is the major risk factor for the development of glaucoma [5]. Currently, the only means of clinically lowering IOP entails the use of daily eye drops and/ or surgery. Most glaucoma patients are elderly and compliance in these individuals, who often require additional medications, is poor [6].

The tissue responsible for maintaining physiological IOP is the trabecular meshwork (TM). Located at the iridocorneal angle, this tissue offers mechanical resistance to the flow of aqueous humor and uses it to regulate IOP. Targeting TM cells with IOP reducing genes [7–10] would allow infrequent (or sole) administrations and would be a highly appealing alternative for treating glaucoma by gene therapy.

Injections of recombinant viruses into the anterior chamber of the eye preferably deliver genes to the TM. Adenoviruses highly transduce the TM of different animal species [11–15], as well as perfused postmortem human eyes [16, 17]. Adenoviruses are able to achieve therapeutic IOP reduction in eyes from perfused human/primates, mice, and sheep [18–21] but the effect is short-term.

Single-stranded DNA adeno-associated viruses (ssAAV) provide instead, long-term expression and low immunogenic profile. Although ssAAV have been preferred for gene therapy of posterior segment eye diseases [22], we have shown that these viruses are unable to transduce the anterior segment TM [23]. Instead, the double-stranded self-complementary form dsAAV (scAAV) transduces the TM very efficiently [23]. In nonhuman primates, a single intracameral injection of scAAV2.CMV.GFP showed noninvasive gonioscopy fluorescence in the TM for 2 years [24]. Transduction to this tissue was extended functionally, and scAAV2 carrying either dominant-negative RhoA or inducible MMP1 therapeutic genes, effectively reduced IOP of rats and sheep in two models of elevated IOP [25, 26].

There is good evidence that ssAAV from different serotypes are cell type and species-specific [27–30], and that transduction efficiencies/tropisms in rodents do not directly translate to nonhuman primates [31]. It is then highly important that comparative evaluations be performed in the human relevant tissue. In the TM, efficiencies of other than scAAV2, or its capsid mutants have been scarcely studied. In mice, scAAV2.smCBA.GFP’s transduction was low and increased with single and triple capsid mutants [32]. An scAAV8.smCBA.GFP with a different single capsid mutation exhibited lower transduction than serotype 2 [32]. In rats, scAAV.CMV.GFP serotype 2’s transduction was detected early and was distantly followed by serotype 8 [33]. At 2–3 months, a highest serotype 2 was followed by serotypes 8, 1, and 5. In the TM of porcine perfused anterior segment organ cultures, serotype 6 was highest, closely followed by 2 and distantly by 5, while 1, 8, and 9 were undetectable [34]. Recently, a new ssAAV, Anc80L65, generated by in silico reconstruction of the virus evolutionary lineage, transduced the TM of mice with higher efficiency than the scAAV2 at 1 month, but this effect was reversed in transformed human TM cells at an earlier time [35].

The transduction efficiency of a given serotype is determined by the efficiency of each of the different steps in the AAV life cycle. With the intent of developing an optimal gene transfer vector for the human TM, in this study we assessed the contribution of several conditions to the tropism of the human tissue. We first performed a quantified transduction with seven AAV serotypes (serotypes 1, 2, 2.5, 5, 6, 8, and 9) in both, their ss and ds forms in primary human trabecular meshwork (HTM) cells. To investigate genetic background influence in the different transduction of the serotypes and assess an individual variation, we compared their transduction efficiency in primary HTM cells independently derived from four single individuals. To further improve transduction, we examined the effect of single and triple mutation capsid mutants of the highest transducing serotype. Given the functional relevance of the specific architecture of the TM system, we assessed the distribution of the transduction by perfusing anterior segment organ cultures from human postmortem donors with the highest transducing vectors. In addition, in view of the lack of transduction of the ssAAV form in all serotypes assayed, and on early observations that the lack of ssAAV2 transduction was not due to its inability to enter the TM cell [23], we quantified and directly compared the number of viral genomes (vg) entering the cell under identical infection conditions of pairs of ssAAV/scAAV of the same serotype. Our study brings up the first systematic analysis of AAV serotypes in the human relevant tissue for the development of glaucoma. It also brings out the novel finding that despite of the same biological composition of the capsid, the packaging of an ss virus can be a determinant of its entry efficiency.

## Results

### Transduction profiles of ssAAV and scAAV serotypes in primary human trabecular meshwork cells from a single individual

For this study, we first determined efficiencies on a single individual, and then continued to examine whether the found differences were general, or individual-dependent (see below).

**Fig. 1:**
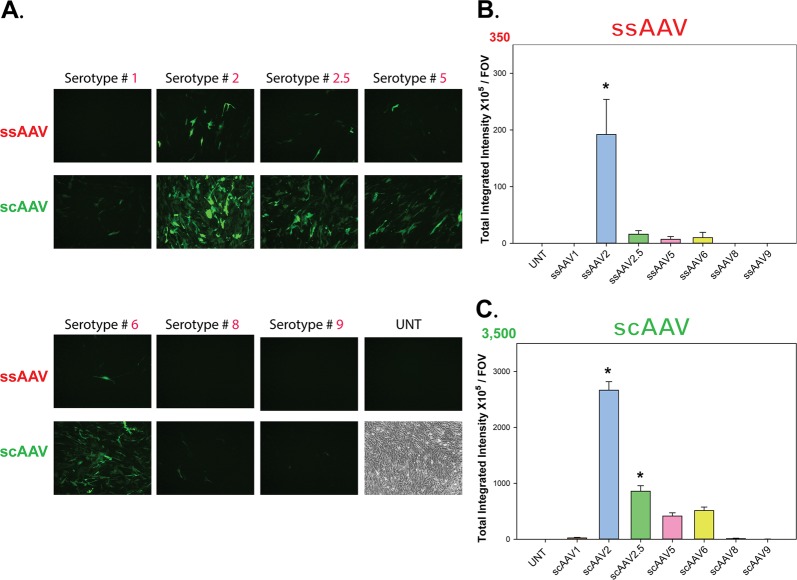
Transduction of seven ssAAV and scAAV serotypes on primary human trabecular meshwork (HTM) cells from a single individual. Subconfluent HTM-210 cells in 12-well plates were infected in parallel with 6 × 10^9^ viral particles of ss and ds forms of AAV.GFP serotypes. Images were captured at the same exposure with a monochrome camera and cellSens software at 7 days post infection. Total integrated intensity of fluorescence was quantified on an average of 8–10 FOV (14,000 cells) using MetaMorph imaging software. **a** Representative fluorescent images of each serotype transduction. **b** Quantification of fluorescent intensity of the ssAAV serotypes *p *<* 0.043. **c** Quantification of fluorescence intensity of the scAAV serotypes. ssAAV serotypes barely transduced the HTM cells while some of their corresponding scAAV were about 10× more efficient (note different scales). Serotype scAAV2 showed the highest intensity followed distantly by sc2.5, sc6, and sc5. Transduction of sc1, sc8, and sc9 was negligible. These results indicate that the human trabecular meshwork tissue is highly refractory to transduction by AAV serotypes carrying ss DNA. Serotype 2 is by far the most efficient serotype to transduce this human cell type. *p *<* 0.01. Original magnification: ×100

An identical CMV-eGFP DNA cassette was packaged in both forms with AAV capsids from serotypes 1, 2, 2.5, 5, 6, 8, and 9 and assayed on primary HTM cells from a 70-year-old Caucasian male (Ind #1; HTM-210). Wells were infected with ssAAV/scAAV serotypes in parallel using 6.0 × 10^9^ viral particles (estimated multiplicity of infection (moi) 20,000 vg/cell) and quantitated for GFP fluorescence expression from 72 h to 7 days using MetaMorph (Olympus) as indicated in “Materials and methods” section. Results are summarized in Fig. 1. HTM cells infected with the seven ssAAV serotypes had very low to negligible GFP expression, indicating that the low efficiency of the ss form previously observed in serotype 2 [23] did extend to all ssAAV serotypes assayed. In contrast, HTM cells infected with the corresponding scAAV serotypes exhibit a much higher efficiency (Fig. 1a).

Despite the very low efficiency of the ssAAV form, a clear rank could be observed. ssAAV2 (ss2) transduction was 13.2× higher than ss2.5, and 21.1–30.1× higher than ss6 and ss5 (*p* *=* 0.043 between ss2 and ss2.5). The differences among ss2.5, ss6, and ss5 were less than 2.3x and were not statistically significant (*p* *=* 0.27 between ss2.5 and ss5). At the same threshold, no fluorescence was detected for ss1, ss8, and ss9 (Fig. 1b).

The levels of transduction of the scAAV viruses were about 10x higher (Fig. 1c). The ranking of efficiency was maintained in the ds viruses, showing scAAV2(sc2)> sc2.5> sc6> sc5> sc1> sc8> sc9. The differences in transduction between sc2 and the second group of less positive viruses were however lower. Thus, sc2 was just 3.1× higher than sc2.5 (*p* *=* 3.9e−8), and 5.1–6.4× higher than sc6 and sc5 (*p* = 7.6e−10 and 2.0e−11, respectively). Also, varying from the ssAAV, the difference between sc2.5 and either sc6 or sc5, was statistically significant (*p* = 0.01 and 7.2e−4, respectively). The difference between sc6 and sc5 though, was not significant (*p* = 0.26). In addition, a very weak, albeit detectable fluorescence was observed in sc1, and an occasionally green cell was observed in sc8, all at the same imaging capture condition exposures (Fig. 1c). No expressing cells were seen in HTM cells infected with sc9 at the same threshold (Fig. 1a).

Altogether these results show that the transduction efficiency of all scAAV serotypes tested is markedly greater than that of their corresponding ssAAV forms in HTM cells. In addition, although the rank of transduction of ssAAV and scAAV serotypes was the same, the transduction ratio among the serotypes in each of the two forms was not, suggesting perhaps that the mechanism of transduction in the HTM cells might not be determined exclusively by the conformation of the genome.

### Internalization, entry, and transduction comparison of highest efficiency serotypes

To next investigate whether the transduction differences observed between ssAAV/scAAV were due to a different entry efficiency of the viral forms, we quantified the number of vg at 2 h (internalization) and 24 h (entry) on the same Ind #1 cells. ssAAV/scAAV infections were always conducted in parallel and cells were exhaustively washed prior to harvesting the intracellular DNA (viral plus genomic). A mock well without cells was treated with the same number of viral particles and same volume of media to control for the potential carryover of viruses during the extraction procedure. Quantification of vg and number of cells was conducted by TaqManPCR using GFP (located at 809 bp from the mutant-Inverted Terminal Repeat, mt-ITR) and human single-copy gene (RPPH1) probes as detailed in “Materials and methods” section.

**Fig. 2:**
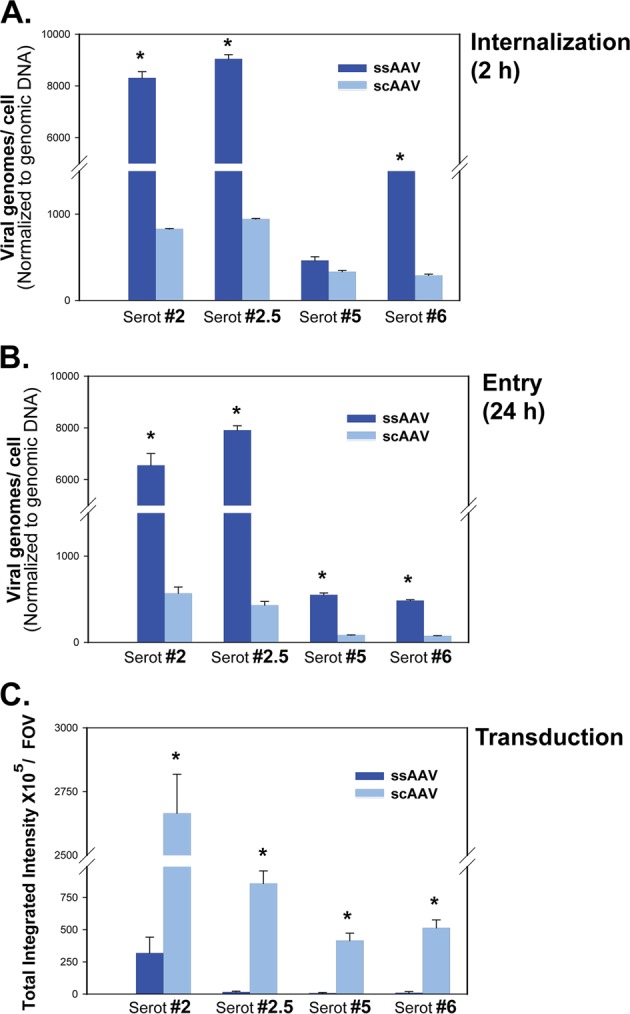
Comparative analysis of internalization, entry, and transduction of serotypes 2, 2.5, 5, and 6 in the same individual. **a **Internalizations graphs expressing vg/cell at 2 h post infection (1 h wet ice, 1 h 37 °C) of subconfluent HTM-210 cells infected in parallel with 6 × 10^9^ viral particles of ss and ds serotypes. *p *<* 0.001. **b** Cell entry graphs expressing vg/cell at 24 h post infection of the same four serotypes. *p *<* 0.002. **c** Total integrated intensity transduction graphs of the same four serotypes at 7 days post infection (reanalyzed from Fig. 1). *p *<* 0.001. The ssAAV showed a considerably greater internalization/ entry efficiency than the ds scAAV in the HTM cells. Transduction efficiencies for ssAAV/scAAV were reversed. These results indicate that in this specific cell type (primary HTM), the transduction advantage of scAAV over ssAAV overcomes the shortage of intracellular viral genomes

In every case, despite the low to no transduction of the ssAAV form, the cell internalization and entry of each ss serotype was significantly higher than that of the corresponding ds, scAAV (Fig. 2a, b). At 2 h post infection we found that the ratios of internalized ssAAV/scAAV vg were 10.0×, 9.5×, 1.4×, and 5.8× for serotypes 2, 2.5, 5, and 6, respectively (*p* *<* 1e−3) (Fig. 2a). After 24 h, the viral entry data exhibited the same profile with ratios of ssAAV/scAAV somewhat higher of 11.5×, 18.4×, 6.6×, and 6.6× for the same serotypes 2, 2.5, 5, and 6 (*p* *<* 2e−3) (Fig. 2b). The total intracellular ssAAV vg at 24 h was markedly higher than those of their corresponding scAAV. Serotypes 1, 8, and 9 exhibited minimal to none cell entry (data not shown).

Transduction efficiencies for ssAAV/scAAV were instead, reversed. The integrated fluorescence intensity (transgene expression) of the ds form was considerably, and significantly higher than that of the ss (Fig. 2c). Expression of scAAV versus ssAAV was 8.4×, 53.7×, 59.3×, and 51.5× higher for serotypes 2, 2.5, 5, and 6 respectively (*p* *<* 1.3e−3).

To address whether the quantified lower viral entry of the ds could had been the result of experimental artifacts due to potential qPCR interfering with the high secondary structure of the mt-ITR, we conducted two type of validation experiments. As a representative example, we used the original ss and ds DNA preparations from the 24 h entry data points of serotypes 2 and 2.5. For the first validation, we eliminated the mt-ITR secondary structure by digesting the viral DNA with *SmaI* (two sites in the mt-ITR of the parental vector [36]) and conducted TaqManPCR amplification before and after the digestion. For serotype 2, we found that, before digestion the number of vg/cell of the ssAAV2 form was 3.6× higher than the number of scAAV2. After digestion with *SmaI*, the ratio of the ss/ds was not changed, with vg/cell values of 3.2× higher in the ssAAV2. Serotype 2.5 showed an ss/ds ratio of 5.5× and 4.1× respectively before and after the treatment with the enzyme. For the second validation, we had the ss and ds AAV2 DNAs independently qPCR quantified for vg at the University of North Carolina (UNC) vector core facility, which uses an ITR probe instead of the GFP one used in our laboratory (see “Materials and methods” section). Their independent results showed that the normalized intracellular number of vg of the ssAAV2 were 3.7× higher than those of scAAV2.

Altogether these findings indicate that the ssAAV forms not only successfully enter the HTM cells but do so at a markedly higher efficiency than those of the scAAV ones. Albeit the extent of the ss increase was reduced in the validation older samples (probably due to different DNA stabilities during the one year storage at −20 °C), the higher entrance obtained after enzyme digestion plus the use of different probes, validates, and reinforces the finding. The results therefore reveal that, in this primary cell type, the high transduction of scAAV over ssAAV overcomes the shortage of available vg. Investigating the cause of this scAAV lower cell entry could lead to the development of more efficient vectors for the TM.

### Capsid mutations of the highest transduction serotype (scAAV2) increased transduction and viral entry

**Fig. 3:**
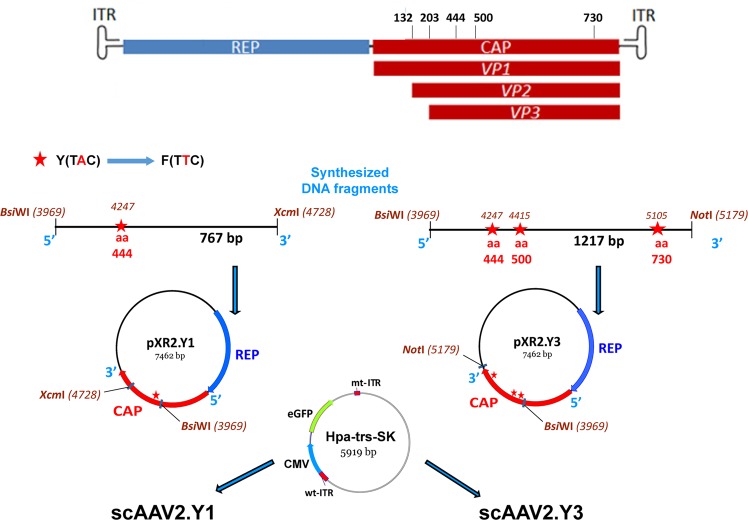
Diagram of the strategy to generate tyrosine capsid mutant plasmids for serotype 2. (Top) Schematic representation of the AAV DNA noting aa locations for the proteins’ start sites and the mutation sites. (Bottom) Diagram of the synthesized DNA fragments (767 and 1217 bp) containing either one or three A to T mutations to yield Y/444, 500 730/ F aa changes. Matching flanking restriction sites allowed swapping the synthetic fragments in the serotype 2 pXR2 rep/cap plasmid at the correct sites (pXR2.Y1 and pXR2.Y3), to produce the corresponding mutated cap proteins. Plasmids were co-transfected with the mt-ITR transgene plasmid Hpa-trs-SK and the Ad helper (data not shown) to generate scAAV2.Y1 and scAAV2.Y3

ssAAV trafficking studies had shown that capsid tyrosine phosphorylation was a signal for proteasome degradation [37], and that capsid Y to F mutations resulted in higher transduction efficiencies [38–40]. To next test the effect of such mutations in TM’s transduction we constructed two sc2 mutant plasmids, containing either single (Y444F) or triple (Y444, 500, 730F) substitutions in the cap gene. The strategy is shown in Fig. 3. DNA fragments 767 and 1217 bp, containing the pXR2 sequence between two single restriction enzyme cutters (5′ *Bsi*WI-*Xcm*I 3′ and 5′ *Bsi*WI-*Not*I 3′), were synthesized carrying either one or three A to T mutations at the selected residues. Synthetic fragments were swapped in the parent AAV2 cap/rep plasmid pXR2 and used to generate scAAV2.Y1 (sc2.Y1) and sc2.Y3 viruses using the mt-ITR transgene plasmid Hpa-trs-SK [36]. An ss2.Y1 was additionally generated using the ITR plasmid pTRUF-CMV-GFP [41].

**Fig. 4:**
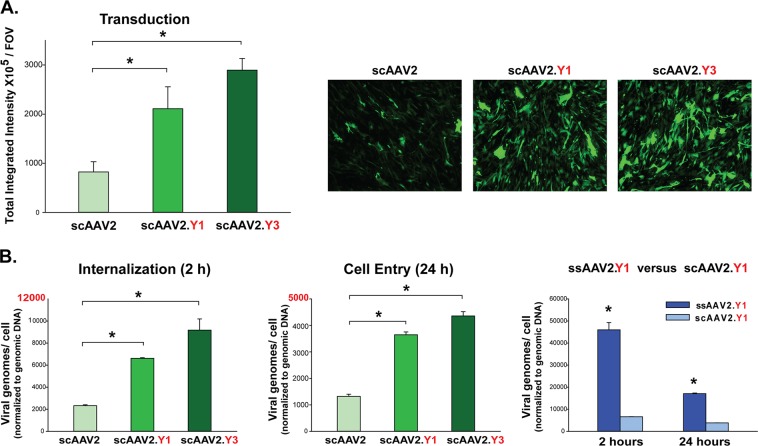
Transduction and entry comparison of capsid mutated scAAV2 viruses. Primary HTM-216 cells in 12-well plates were infected at subconfluency with 6 × 10^9^ viral particles of sc2, sc2.Y1, and sc2.Y3. Fluorescence intensity (transduction) was assessed at 72 h post infection. Intracellular DNA was harvested at 2 and 24 h. **a** Transduction quantification and representative images showing a significant increase between sc2.Y1 or sc2.Y3 and sc2, of 2.6× and 3.5× respectively. *p *<* 0.05. Original magnification: ×100. (**b**, left and center) vg/cell quantification at 2 and 24 h (internalization and cell entry) of sc2, sc2.Y1, and sc2.Y3 showing significant increases similar to those seen in transduction. Number of intracellular vg/cell at 24 h is much lower than that at 2 h (note different scale). *p *<* 0.02. (**b**, right) Comparison analysis of the internalization and entry parameters of an ss2.Y1 with its corresponding sc2.Y1 showing the significant entry increase of the single-stranded form. *p *<* 0.001. These results indicate an enhanced transduction and entry of the mutated capsid virus, albeit non proportional with the number of aa mutations. They also showed that viral particles carrying the mutant capsids are protected from very early but also appear to suffer from some degradation during intracellular trafficking

In HTM cells from Ind #4 (Caucasian female 57 years old), a single tyrosine substitution, sc2.Y1, increased transduction efficiency 2.6× (*p* = 0.047) (Fig. 4a). Transduction of the virus with three mutations, sc2.Y3, was 1.4× insignificantly higher than sc2.Y1 (*p* = 0.16) albeit 3.5× significantly higher than the nonmutated sc2 (*p* = 3.8e−4). The entry of the capsid mutants was also higher than the nonmutated sc2 and their entry profile mimicked that of transduction (Fig. 4b). At 2 h, sc2.Y1 infected cells had 2× more vg than sc2 (*p* = 2.8e−3), and sc2.Y3 had 1.4× more than sc2.Y1 (statistically not significant; *p* = 0.13) but 3.9× more vg than the sc2 (statistically significant; *p* = 0.02) (Fig. 4b, left). At 24 h, sc2.Y1 had 2.8× more molecules than sc2 (*p* = 2.0e-5), and sc2.Y3 had 1.7× more vg than sc2.Y1 (statistically not significant; *p* = 0.13) but 3.3× more vg than sc2 (statistically significant; *p* = 4.6e-6) (Fig. 4b, center). This finding indicates that there is protection from the proteasome pathway in the HTM and the protection occurs very early, right at the internalization step.

We further observed that the total number of vg at 24 h was lower than that at 2 h (notice scale difference on Fig. 4b, left and center graphs), indicating some additional degradation during intracellular trafficking. Lastly, to investigate ss entry characteristics in the tyrosine mutants we generated an ss2.Y1. As it had occurred with the nonmutated viruses, the entry of the ss mutant was higher than the ds showing 6.9x and 3.5x increases at 2 and 24 h, respectively (*p* *<* 0.001) (Fig. 4b, right).

Altogether, these results indicate that the human TM-high transgene expressing virus sc2.Y3 becomes a favorite vector for developing a treatment of elevated IOP by gene therapy. The data also continues to support the notion that in this cell type, the ssAAV binds and enters the cells at a higher concentration than the ds form.

### Transduction and entry of scAAV serotypes in primary human TM cells are individual-dependent

**Fig. 5:**
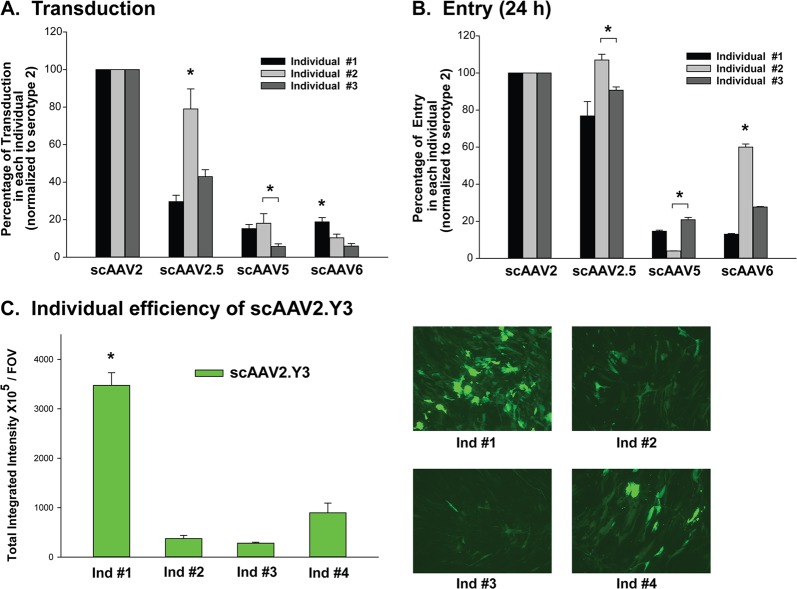
Efficiency of scAAV serotypes in different individuals. Primary HTM cells were generated and characterized from a Caucasian male 70 years old (Ind #1); a Caucasian female 62 years old (Ind #2), a Caucasian male 90 years old (Ind #3), and a Caucasian female 57 years old (Ind #4). Cells from the four individuals were infected with the indicated serotypes, assayed for transduction at 72 h post infection and for cellular entry of vg/cell at 24 h. **a** Percentage of transduction of highest nonmutated serotype sc2 in three individuals showing distinct individual profiles. *p *<* 0.02. **b** Percentage of intracellular vg/cell normalized to highest nonmutated serotype sc2 in three individuals showing distinct individual profiles. *p *<* 0.05. **c** Transduction of mutant sc2.Y3 on four individuals showing marked transduction response in Ind #1. *p *<* 0.02. Original magnification: ×100. These results indicate that there is an individual constituent, which does not seem to greatly affect the rank of serotypes efficiency but that it has a marked influence on the intensity of the response

In humans, genetic individual variability plus race, gender and/or age contributes to their responses to drugs, stress and many disease inducers. To this end, our laboratory had shown that the TM harbors a set of genes which respond differently to IOP among individuals (e.g., genes highest responders in one individual while unresponsive in others), plus other set of genes which are general responders [8]. To address whether the distinct sc serotypes’ efficiency was influenced by an individual component, we took advantage of the fact that each of our primary HTM cells has been generated from the eye(s) of a single individual. We first compared four transducing scAAV serotypes in three distinct individuals, and then compared the highest mutant sc2.Y3, in the same three cell lines plus an additional one. Transduction and entry of the serotypes in Ind #1 (Caucasian male 70 years old), Ind #2 (Caucasian female 62 years old), and Ind #3 (Caucasian male 90 years old) is represented as percentage normalized to their own sc2 for clarity and visibility (Fig. 5a, b). We found that upon normalization to sc2, transduction of each serotype was different in each individual (Fig. 5a). Thus, sc2.5 and sc5 were higher in Ind #2 than in Ind #1 or Ind #3 (*p* *=* 0.006 and 0.003 for 2.5; *p* *=* 0.7 and 0.02 for 5, respectively) while sc6 was higher in Ind #1 than in Ind #2 or Ind #3 (*p* *=* 0.02 and 4e−5, respectively). For the entry at 24 h, the normalized profile of sc2.5 was similar to that of transduction, that is, higher in Ind #2 than in Ind #1 or Ind #3 (*p* *=* 0.06 and 0.05). However, sc5 was higher in Ind #3 than in Ind #1 or Ind #2 (*p* *=* 0.06 and 0.01), while sc6 was higher in Ind #2 than in Ind #1 or Ind #3 (*p* *=* 0.001 and 0.003).

To next compare the absolute integrated intensity transduction of a given serotype in all individuals, we used the highest efficiency mutant, sc2.Y3 (the transduction and integrated intensity will describe below). We infected cells from the above individuals plus those of a 57-year-old Caucasian female (Ind #4). Integrated intensity of ten field of views (FOV) per well were averaged and plotted in Fig. 5c. The highest transduction in Ind #1 showed a 3.9× increase over the next in line, Ind #4 (*p* *=* 0.02). Transductions among Ind #4, Ind #2, and Ind #3 were not significantly different (*p* *>* 0.09) (Fig. 5c). Altogether, these results indicate that scAAV serotypes have different transduction efficiencies in different human individuals. However, although the relative transduction and entry of each serotype varies with the individual, the two highest serotypes in each individual remained the same.

### scAAV2 single and triple mutant transduction localized to all layers of the human trabecular meshwork in perfused eyes from postmortem human donors

**Fig. 6:**
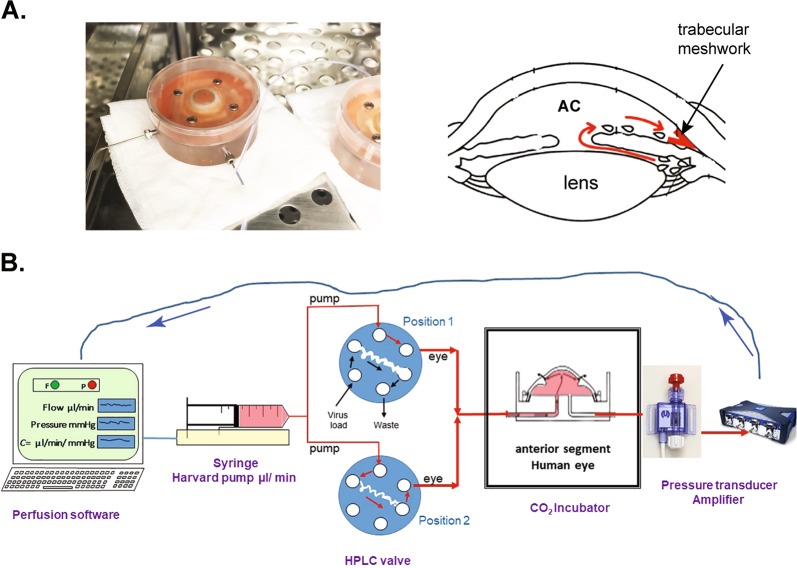
Composite image depicting the anterior segment organ culture perfusion of human eyes from postmortem donors. (**a**, left) Actual human eye from a postmortem donor mounted on a custom-made chamber showing entrance and exit of the perfusing media. (**a**, right) Schematic representation of the flow of aqueous humor in the eye showing the exit through the trabecular meshwork. **b** Schematic representation of the full human eye perfusion system which mimics gene delivery to the actual human eye. A UNC custom-made computer program controls the rate of the perfusion pumps carrying the infusion syringes filled with media. HPLC valves equipped with a 20 µl virus loaded loop are intercalated between the syringes and the incoming cannula of the eye chamber to command time of viral delivery. Position 1 will direct perfusion media to the eye, while position 2 will direct delivery of the virus loaded in the loop to the eye at the programed time; the system is reversed to position 1 to resume media perfusion post delivery. Second eye cannula from the eye chamber is connected to a pressure transducer, amplifier, and back to the computer (drawn line) for recording of IOP values and confirmation of physiological conditions

**Fig. 7:**
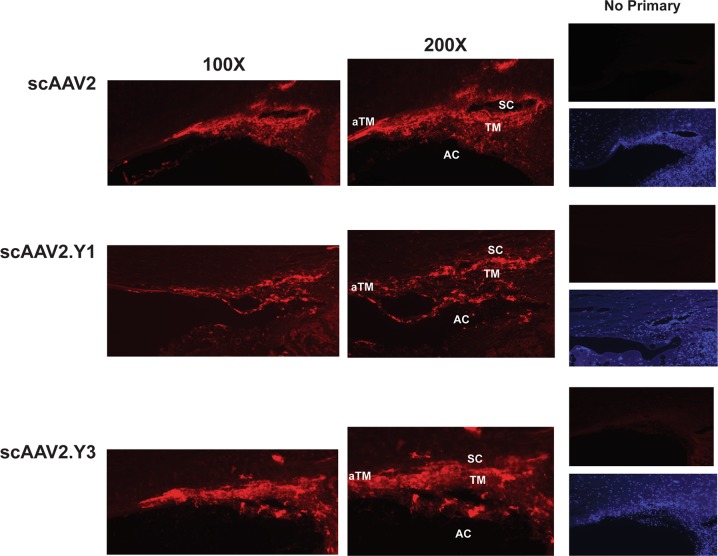
Perfusion of three pairs of postmortem human eyes. Eyes, 12–27 h postmortem, were mounted, perfused for 24 h and infected with the indicated serotypes through the HPLC system (*n* = 6 eyes, 2 per serotype). Perfusion continued for 4–6 days post infection and quadrants from the anterior segment were fixed, embedded and processed for immunocytochemistry. Shown are representative 10 µm cryosections of tissues infected with the indicated serotypes, stained with a chicken anti-GFP antibody (1:500) and counterstained with goat anti-chicken Alexa Fluor 594 (1:500). Control sections (*right* panels) show corresponding sections processed without the primary antibodies and counterstained with DAPI for morphology visualization. These images confirm the original distribution of scAAV2 in the human trabecular meshwork tissue and indicate that the new vectors containing the mutated capsids conserve the location and distribution of their carried transgene. Based on these findings, the scAAV2.Y3 vector appears as the best candidate for gene therapy of glaucoma. aTM anterior trabecular meshwork, TM trabecular meshwork, SC Schlemm’s canal, AC anterior chamber

The TM of the human eye exhibits a specialized architecture designed to regulate the flow of aqueous humor. It consists of several cell types arranged in layers according to their function. While primary cultures include all cell types, it is not possible to distinguish whether all cell types are transduced. Fortunately, human eyes can be procured a few hours postmortem and their whole anterior segments set in perfusion cultures up to 4 weeks [42]. The system mimics the physiological flow of aqueous humor and the IOP resulting from the resistance to the flow by the TM (Fig. 6). Using these perfused organ cultures, we previously reported that an sc2 vector transduced all cell types of the human TM [23]. Here, we sought to determine whether capsid mutated viruses sc2.Y1 and sc.Y3 conserved the local transduction distribution in the intact tissue. For this study, we used three pairs of normal, nonglaucomatous human eyes, received 12 h and 27 h postmortem and prepared them as detailed in “Materials and methods” section. Anterior segments were perfused at 3 µl/min for 24 h to revive and equilibrate the tissue (Fig. 6). Average outflow facilities values (*C* *=* Pressure/Flow) at baseline for the first pair (Caucasian male 69 years old) were 0.14 and 0.11 µl/min/mmHg for the right and left eyes, respectively. For the second pair (Caucasian male 75 years old), the *C* values were 0.15 and 0.21 µl/min/mmHg, and for the third pair (Caucasian female 62 years old) 0.14 and 0.05 µl/min/mmHg. These values are within the range of the physiological *C* of the human eye [43]. After equilibration, loops were loaded with 2–10 × 10^10^ vg of the corresponding virus and delivered to the anterior chamber as indicated in “Materials and methods” section. Four to six days post delivery, tissues were fixed, sectioned and stained with a chicken anti-GFP antibody. Figure 7 shows that delivery of the transgene occurred to all different layers of the human TM. Staining was observed also in the inner and outer walls of the Schlemm’s canal and on the anterior TM, area known to harbor the TM stem cells. These results indicate that the mutated capsids in the serotype 2 did not affect their distribution and ability to transduce all distinct cell types of this specialized tissue. Therefore, this finding together with their higher transduction efficiency makes them good candidates for gene therapy of glaucoma.

## Discussion

Clinical management of elevated IOP remains one of the biggest challenges for the treatment of glaucoma. The search for treatments other than the daily administration of eye drops or surgery, places gene therapy as a very attractive and realistic possibility to treat this common disease. The extensive knowledge about the physiology, biochemistry, and molecular biology of the responsible TM tissue, its accessibility, and the potential of investigating gene delivery strategies using the actual human tissue are unique advantages. We and others had previously investigated the use of viral gene transfer to the TM (reviews in [44–46]). However, very little has been published regarding the ability of using ss- and scAAV and its different serotypes to transduce this eye tissue, and even a lower number of studies are available, which use the human tissue. Because of our unique resources (human primary HTM cell line collection and perfused postmortem organ cultures), we aimed to fill this gap by examining the transduction efficiency and cellular entry of seven serotypes (1, 2, 2.5, 5, 6, 8, 9) (six common natural occurring AAV isolates plus one hybrid serotype) [31] in both, the ss and ds forms.

We found that, as it had occurred for ss2 [23], none of the other six ss serotypes induced a significant transduction. In contrast, four sc serotypes (2, 2.5, 5, and 6) transduced the human TM at about 10× higher efficiency (Fig. 1b). The efficiencies among the sc transducing serotypes were different. Serotype sc2 had the highest transducing values in this cell type, distantly followed by serotypes 2.5, 6, and 5. Transduction of serotypes 1, 8, and 9 was negligible. These findings about the severe compromised transduction of all ss and the high transduction of the ds forms restate the concept that TM barriers of ssAAV transduction are cell-type specific and rather than the capsid, appear to involve host restriction factors.

Comparative analysis of the biology of AAV serotypes and the TM gives further insights. Serotype 2 had the highest transduction efficiency in the TM. It is well-established that ss2 uses heparan sulfate (HS) as a primary receptor, and integrins αVβ5/α5β1 as coreceptors [31]. These molecules are well-known to TM biology. The HS receptor was early determined to be a part of the relevant glycosaminoglycans of the tissue [47] and a contributor to the filtration barrier to the aqueous humor flow through the TM [48]. More recently, it has been shown that exosomes involved in the regulation of TM extracellular matrix turnover and opening of outflow channels, need to bind to HS to carry their function [49]. Further, ss2 coreceptors αVβ5/α5β1 are among the integrins found heavily distributed on the TM cells, especially on cells of the corneoscleral region. These integrins bind to RGD domains of key TM regulatory proteins and are the subject of intensive investigation [50]. Thus, it is not surprising that serotype 2 is the best equipped to enter the TM. But even these receptors’ advantage is not enough to overcome the ss2 barrier to transduce the TM. To this end, it is worth to note that an ss2 engineered to contain the RGD domain in its VP3 gene [51] was unable to transduce the TM, albeit it was detected intracellularly [23].

Hybrid serotype sc2.5, although distant, was the second most efficient in the HTM cells. ss2.5 was constructed by inserting five selected amino acids from ss1 into ss2 to increase the latter’s muscle tropism while retaining the HS receptor [52]. This chimeric vector also showed superior transduction than ss2 in the mice retina [53]. However, the ds form, sc2.5 had significantly lower transduction than sc2 in the TM. This marked reduction indicates that the five amino acids of ss1 might be part of the reason why the sc1 serotype is so negative in HTM cells. Interestingly, atomic structure determination of ss2.5 revealed that the virion retains the conformation of ss1 [54], which may be also influencing its lack of transduction in the HTM cells.

Interestingly, sc6’s transduction was next to sc2 and sc2.5. The ss6 virus belongs to the same clade than ss1 and its capsid proteins differ in just six amino acids [54, 55], which are thought to affect receptor binding and postentry functions [54]. The two serotypes have almost identical serology [56]. However ss6, in addition to binding to ss1 receptor c1α2,3 and α2,6 N-linked sialic acids, binds to the HS receptor, while ss1 uses only the sialic glycans [54, 56]. This could be the reason as to why, sc6 has a moderate, always measurable transduction in the TM while sc1 transduction is barely detected or not detected at all. Although TM early studies had reported the presence of sialic receptors in the Schlemm’s canal cells [57], these cells from the one-cell layer of the canal are difficult to grow and comprise only a fraction of the general primary HTM cultures, which could explain the observed lower transduction. Interestingly, our early studies using the entire TM tissue in perfused porcine organ cultures showed sc6 to be the highest transduced serotype, followed by sc2 [34].

Serotype ss5 is one of the most divergent serotypes with just a 56% cap sequence similarity with ss2 but using the same sialic receptor as 1 and 6 [58]. Curiously, in the HTM cells sc5 showed entry and transduction very similar to that of sc6, their ranks alternating but not significantly. Serotypes sc8 and sc9’s transduction, despite been preferred for liver and heart [59–61] was negligible in the TM. The high refractory nature of HTM cells to these two serotypes is puzzling. Both ss8 and ss9 use the laminin receptor [62], which binds laminin, and laminin is another of the well-characterized components of the juxtacanalicular region of the TM [63]. Thus, a different restriction factor seems to be playing a role in preventing their entry and transduction. Factors affecting transduction are continually being uncovered. A recent publication combining atomic force microscopy (AFM) with biochemical experiments showed that physical properties and stability of the ssAAV particles have an influence on genome ejection pathways which in turn, can also contribute to different serotypes’ transduction efficiencies [64]. At the present time we do not know whether the recently discovered AAVR receptor [65] is present in the TM, or whether its presence would add to the efficiency of the current serotypes. But judging by its similar effect on all serotypes studied here one would expect that such receptor would not change their hierarchy in this eye tissue.

Perhaps the most striking finding of our studies was the fact that in these HTM cells, the ssAAV serotypes despite the nontransduction, have higher internalization and entry efficiency than their counterpart serotypes in the ds form. Our previous studies had shown that a nontransducing, ss2 was able to enter the cell [23]. Here, using more advanced quantification technology, we found that for every serotype, the number of intracellular ss vg was between 4 and 10× higher that the vg number of the corresponding ds. The marked difference between ss and ds was observed already at the 2 h internalization point and slightly increased at 24 h when the virus has reached the nucleus [66]. The reason for this phenomenon is currently unknown. Recently, an AFM study found that the cargo of the particle led to measurable distinct mechanical properties of the ss and ds virions and that scAAV vectors were the most resilient ones to external compression [67]. It would be intriguing to think that such mechanical characteristics of particles with different genomic cargo could influence the attachment and entry of the virus.

Putting together these findings, it would of great interest to identify and then inhibit the specific host restriction factor that impedes the transduction of ssAAV in the TM. Inhibition of such factor would allow the use of the ssAAV platform in the TM, which considering their favorable entry could result in a considerable higher transduction. To this end, the ssAAV Anc80L65, selected from phylogenetic analysis and reconstruction of capsid sequences from ancestral serotypes 1, 2, 8, and 9 [68], could turn out to be a good choice other than for allowing increase in the size of the cargo.

Viral capsids of sc2 mutated at aa 444 (one Y change) or at aa 444, 500, 730 (three Y changes), led to significantly increased transduction. The increase of the triple mutant over the single one was smaller than that reported in other studies [69] but had been noticed on mice using the same Y mutations with a different promoter [32], suggesting it might be specific for the TM. Importantly, an increased number of intracellular sc2.Y1 and sc2.Y3 vg over sc2 was observed already at 2 h post infection, indicating that the endosomes capsid mutants’ protection occurred very early, practically at the time of internalization. In addition, the Y-mutants conserved the higher ss/ds entry observed with the nonmutated capsids. Together, these findings helped to define the most optimized vector to date for human TM gene therapy (sc2.Y3).

Our study also revealed that there is an individual component to the transduction efficiency of each serotype. The finding is not surprising since the human genetic profile of single individuals, in contrast to selectively bred animals, is quite diverse [8]. In the future, it could be interesting to potentially use differentiated TM stem cells to pretest individual’s susceptibility to serotypes prior to a gene therapy application.

Finally, the most efficient vectors were able to transduce all cells from all layers (uveal, corneoscleral, juxtacanalicular, and Schlemm’s canal cells) of this complex tissue on perfused human eyes from postmortem donors. The purpose here was to investigate distribution of the transgene delivery rather than the total extent of transduction, which is a difficult parameter to evaluate due to the segmental outflow of the aqueous humor (reviewed in reference [70]). Because in these cultures the TM maintains the physiological architecture of the human eye, the results of these experiments serve to validate a potential future development of gene transfer for treatment of this tissue.

In summary, our report provides the first thorough study of transduction efficiency of seven AAV serotypes in both their ss and ds forms, on primary HTM cells and perfused cultures from the human eye. We have identified the serotype 2 in the ds form and with a triple capsid mutation as the preferred vector to deliver genes to the TM and attempt to control elevated IOP. We further evaluated the entry parameters of these vectors and surprisingly found that the ss form, despite its inability to transduce, enters the cells at four to ten times the efficiency than the ds. We thus, believe the results of our studies provide the confidence on the translational step needed to select the optimal serotype to conduct gene transfer to the human TM for the treatment of glaucoma.

## Materials and methods

### Primary culture of human trabecular meshwork cells

Primary HTM cell lines were generated from single residual cornea rims of patients after surgical corneal transplants at the UNC Eye Clinic. The TM was isolated from surrounding tissue under a dissecting microscope by making incisions both anterior and posterior to the meshwork and removing it using forceps. The tissue was cut into small pieces, carefully attached to the bottom of a 2% porcine gelatin-coated (Sigma/Aldrich, St. Louis, MO, USA, cat #G2500) 35 mm dish, and coverslipped with a drop of MEM Richter’s Modification medium (Modified IMEM, Gibco/ThermoFisher, Waltham, MA, USA cat # A1048901) supplemented with 20% fetal bovine serum (FBS, Gibco/ThermoFisher cat #10437-028), 50 µg/ml gentamicin (Gibco/Life Technologies/ThermoFisher cat #15750-060). Cells from these specimens were not treated with enzymes, and were allowed to grow from the explant for 4 weeks changing the media every other day; upon confluency, cells were harvested and stored in liquid nitrogen. When reconstituted, these primary nontransformed cells are grown in IMEM, 10% FBS, gentamicin (complete medium) and subsist for five to six passages. All cells were used at passages 3–5. These outflow pathway cultures comprise all cell types involved in maintaining resistance to flow. That includes cells from the three distinct regions of the TM plus cells lining the Schlemm’s canal. Because most of the cells in these cultures come from the TM, they are commonly referred to as “TM cells”. The cells used in this study were from a 70-year-old Caucasian male (HTM-210, Ind #1), a 62-year-old Caucasian female (HTM-213, Ind #2), a 90-year-old Caucasian male (HTM-195, Ind #3), and a 57-year-old Caucasian female (HTM-216, Ind #4). Each of these individual cell lines was characterized by morphology and by the specific induction of myocilin (MYOC) by dexamethasone (D-8893 Sigma/Aldrich) treatment [71, 72].

### Adeno-associated vectors and production

For this study, seven different AAV serotypes were used. Serotypes 1, 2, 2.5, 5, 6, 8, and 9 [58, 73] carrying the same eGFP transgene driven by the CMV promoter-enhancer were produced in the ss, ssAAV, and ds, scAAV, forms. Viral vectors were manufactured at the UNC Vector Core facility (http://genetherapy.unc.edu/vectorcore). The three recombinant plasmids used for the production of the ssAAV were: pXX6-80, carrying the adenoviral vector helper function genes [74], pTRUF-CMV-eGFP, carrying the eGFP expression cassette in between two wild-type ITR [41, 75, 76], and each of the rep/cap plasmids carrying the AAV Rep2 plus the corresponding serotype-specific cap genes of AAV1, AAV2, AAV2.5, AAV5, AAV6, AAV8, and AAV9 plasmids (pXR1, pXR2, pXR2.5, pXR5, pXR6, pXR8, and pXR9 [27, 52, 56, 77]. All recombinant plasmids were provided by the UNC Vector Core facility. For the generation of the scAAV, the transgene plasmid was substituted by pHpa-trs-SK [36], which contains the same expression cassette plus an additional bovine growth hormone polyadenylation site. In the pHpa-trs-SK, the expression cassette is flanked by one wild-type ITR and one mt-ITR which allows for the generation of the ds DNA [36]. Viral titers were calculated by qPCR at the UNC Vector Core by established procedures [78] and ranged between 0.75–9.5 × 10^12^ vg/ml. Viral high titer stocks were aliquoted upon receiving them at the laboratory and stored at −80 °C. Aliquots were thawed just one to three times before use. Experiments were performed with two to three different viral lots per serotype.

### Plasmids constructions and generation of AAV2 capsid mutants

For the generation of viruses carrying mutated cap 2 genes (see results and Fig. 3), we analyzed pXR2’s restriction site map and selected single cutters flanking nucleotide positions 4247, 4415, and 5105, which form part of the codons encoding tyrosines Y444, Y500, and Y730 in the cap 2 gene (NC_001401.2). Two DNA fragments containing either codon Y444 or codons Y444, Y500, and Y730 were identified in silico between 5′-*Bsi*WI-*Xcm*I-3′ (767 nt) and 5′-*Bsi*WI*-Not*I-3′ (1217 nt) of pXR2. The two fragments with A to T mutations at the positions indicated above were synthesized at Blue Heron (Bothell, WA, USA), inserted into their plasmids pBH.PUCMinusMCS (pBH2 and pBH4) and delivered to us in GC10 competent cells. Transformed bacteria were grown, plasmid DNA purified, and inserted mutations confirmed by sequencing. pBH2 and pBH4 digested fragments (*Bsi*WI-*Xcm*I 767 bp and *Bsi*WI*-Not*I 1217 bp) were purified by gel electrophoresis with low melting agarose (Nusieve GTG or Seakem GTC; Lonza, Allendale, NJ, USA). Isolated fragments were swapped with the corresponding nonmutated ones in pXR2 by ligation to the predigested isolated backbone with a Ready-To-Go T4 DNA ligase kit (Amersham Biosciences, Piscataway, NJ, USA cat #27-0361-01). The resulting DNA was transformed into Sure (Agilent, Santa Clara, CA, USA cat #200238) or DH5α (ThermoFisher, cat #18265017) competent cells to yield plasmids pLR2 (pXR2.Y1) and pLR3 (pXR2.Y3). Transformed bacteria were grown overnight in Fast-Media Amp-LB (InvivoGen, San Diego, CA, USA cat #fas-am-b) and plasmid DNA extracted with EndoFree Plasmid Maxi Kit (Qiagen, Germantown, MD, USA cat #12362) following manufacturer’s recommendations. Purified DNAs were then delivered to the UNC Vector Core for the production of the scAAV2.Y1, scAAV2.Y3, and ssAAV2.Y1 viruses whose titers were determined as indicated above.

### Transduction assays, imaging analysis, and fluorescence quantification in primary human trabecular meshwork cells

For transduction evaluation, primary HTM cells at passages 3–5 were seeded at 40–60% confluency in 12-well plates and grown to subconfluency. Cells were fed 2–4 h prior infection, media changed to 2% FBS and cells were exposed to ssAAV and scAAV in 1 ml for 1 h. After that, complete medium was added and incubation continued from 3 to 7 days. All infections were performed with the same number of vg (6.0 × 10^9^), which resulted in an estimate moi of 20,000 vg/cell. The moi calculation was based on an estimated number of 3.6 × 10^5^ Hela cells/12-well plate at 90% confluency (https://www.thermofisher.com/us/en/home/references/gibco-cell-culture-basics/cell-culture-protocols/cell-culture-useful-numbers.html). Uninfected control wells were run in parallel. For the transduction experiments, a total *n* = 101 wells were infected, *n* = 33 with ssAAV and *n* = 62 with scAAV. There were *n* = 33 wells infected for Ind #1, *n* = 28 for Ind #2, *n* = 23 for Ind #3, and *n* = 4 for Ind #4. Infection with each serotype in the corresponding experiment was at least duplicate.

Assessment of green fluorescence intensity was conducted in an IX71 inverted fluorescence microscope (Olympus, Center Valley, PA, USA) equipped with a monochrome DP80 camera and cellSens software (Olympus). All images were captured at the same nonsaturated exposure time using cellSens. For each well, images were taken from 8 to 10 representative FOV (1.4 mm^2^). Quantification of fluorescence in each field was obtained in the gray scale mode using MetaMorph digital imaging software (Olympus) and using the same threshold value for all wells of a given comparative experimental set. The threshold was determined on the brightest well, and fluorescence levels were expressed as total integrated intensity per FOV. Wells showing no fluorescence at this threshold, were considered as negative transduction. A total 8–10 FOV in our microscope specifications corresponds to a total of about 14,000 cells.

Digital images were arranged with Adobe illustrator CS5 and Photoshop CS software (Adobe Systems, San Jose, CA, USA).

### DNA extraction, TaqManPCR, and quantification of intracellular viral genomes

To assess and quantify intracellular vg at 24 h, primary HTM cells were seeded in 12-well plates and infected under the same culture conditions and moi described above for transduction assays. For cell internalization, subconfluent cells were chilled at 4 °C with cold PBS (Gibco/Life Technologies/ThermoFisher cat #10010023) and placed on ice. Viruses were added to the cells in 1 ml 4 °C PBS and allowed to bind to the cell surface for 1 h. Unbound viruses were washed off with cold PBS and warm medium was added to the cells to stimulate entry. Cells were incubated for an additional hour at 37 °C [79]. After the 2 and 24 h incubation times, wells were exhaustively washed with PBS and harvested for the extraction of total intracellular DNA using a DNeasy Blood and Tissue kit (Qiagen, cat #69504), which yields both, the low MW viral DNA and the high MW human genome DNA (gDNA). Harvesting was conducted by adding 200 µl PBS, 200 µl AL buffer, and 20 µl proteinase K directly to the washed well and scraping the cells with a cell lifter. Extraction continued following manufacture’s recommendations, final columns eluted with 55 µl of AE buffer, and samples saved at −20 °C until ready to assay. Wells containing either infected medium with no cells, or uninfected cells, were run in parallel as controls. For the 24 h entry experiments, a total *n* = 99 wells were infected, *n* = 42 with ssAAV and *n* = 57 with scAAV. There were *n* = 39 wells infected for Ind #1, *n* = 27 for Ind #2, *n* = 18 for Ind #3, and *n* = 15 for Ind #4. Infection with each serotype was on at least duplicate. For the 2 h internalization, a total *n* = 33 wells were infected, *n* = 16 with ssAAV and *n* = 17 with scAAV. There were *n* = 20 wells infected for Ind #1, *n* = 3 for Ind #2, and *n* = 10 for Ind #4. Infection with each serotype in the corresponding experiment was at least duplicate.

Quantifications were performed by real-time TaqManPCR in a StepOne Plus instrument (Applied Biosystems, AB, Foster City, CA, USA). Equal volume aliquots (1 µl) from each DNA sample were used to quantify vg and gDNA. Reactions were performed in duplicate in 20 µl using 10 µl of TaqMan Fast 2X universal PCR master mix (AB cat #4352042), 1 µl of the corresponding probe and 1 µl of template experimental sample DNA. Nontemplate controls were run in parallel. Restriction enzyme digestion with *SmaI* (New England Biolabs, NEB, Ipswich, MA, USA, cat #R0141S) was conducted overnight at room temperature in NEB CutSmart buffer. Prior to TaqManPCR, samples were cleaned up on the DNeasy kit column for enzyme removal and buffer exchange.

For vg quantification, duplicate DNA samples were hybridized to a GFP probe (AB, Mr04097229_mr, cat #4331182) located at 809 bp from the mt-ITR. Quantification was performed against a standard curve generated with 580,000–5.8 molecules (Ct values 19.4–39.6) of a ds pCBh-eGFP plasmid [80], which carries the same enhanced GFP. Levels of ssAAV genomes were corrected by multiplying 2× the number obtained with the ds plasmid. For the gDNA, quantification, duplicate samples were hybridized to a single-copy human gene probe (AB human ribonuclease P RNA component H1, RPPH1; single exon probe Hs03297761_s1 cat #4426961 [25]. Quantification was performed against a standard curve generated with 70–0.3 ng (Ct values 24.1–34.1) of commercial human genomic DNA male (AB cat #360486). To allow comparisons among runs, all GPP runs were analyzed at C_T_ threshold of 0.18 and all RPPH1 runs were analyzed at C_T_ threshold of 0.4. Data are reported as the number of vg molecules per diploid cell. Amount of human genomic DNA (2.15 × 10^12^ MW) is converted to be 140 cells per ng. The total number of vg/cell was used to compare entry efficiency among the ss and ds/sc serotypes.

### Transduction to intact human trabecular meshwork from postmortem human donors

Because of the possibility of reviving the eye anterior segment from donors before 40 h postmortem, perfused organ cultures of these tissues have been traditionally used for molecular and physiological studies of the TM [81–84]. Donors eyes were obtained from the Miracles in Sight Eye Bank (Wiston-Salem, NC, USA) following signed consent of the patients’ families. All procedures were in accordance with the tenets of the Declaration of Helsinki. Whole eyes arrived the laboratory within 12–27 h of death, then dissected at the equator, cleaned away from vitreous, iris and lens, ciliary body removed with iridectomy scissors, and anterior segments mounted on custom-made perfusion chambers as described previously [8, 17, 23, 42, 85]. Perfusion was conducted at constant flow (1.5–4 µl/min) through one of the chamber’s two cannulas with serum-free high glucose DMEM (Gibco/ThermoFisher, cat #11965-092) containing antibiotics using a Harvard microinfusion pump (Harvard Bioscience, South Natick, MA, USA). HPLC pumps (MX7900-000, Rheodyne, Rhonert Park, CA, USA) equipped with a 20 μl loop were intercalated between perfusion syringes and chambers to be able to administer virus without injection through the cornea. All pumps were controlled by a custom-made computer program (Infusion Pump Control Program, UNC Chemistry Department, Electronic Design Facility). Anterior segments were maintained at 37 °C, 5% CO_2_, and perfused for 24 h to establish a stable baseline (Fig. 6). Outflow facilities (*C* *=* Flow/Pressure in µl/min/mmHg) were calculated from pressure recordings and baseline values taken just before the delivery of the viruses. The average outflow facility at baseline for all six eyes in the study was *C* = 0.13 ± 0.02 µl/min/mmHg.

After obtaining a stable baseline, HPLC loops were loaded with sc2, sc2.Y1, and sc2.Y3 viruses (*n* = 2 eyes each), which were delivered inside the chamber by remote control loop injection from the computer. Anterior segments were harvested at 4–6 days post infection and processed for immunohistochemistry.

### Immunohistochemistry

Upon ending the experiment, anterior segments were removed from the chambers, sectioned into quadrants and fixed in 4% paraformaldehyde/PBS for 6 h at 4 °C. Specimens were then immersed in 10% sucrose/PBS for 6 h, transferred to 30% sucrose overnight at 4 °C, and small wedges embedded in optimum cutting temperature compound (Tissue-Tek, Sakura Finetek, Torrance, CA, USA). Meridional 10 µm cryosections were dried at room temperature, rehydrated with DPBS Ca/Mg (Gibco/Life Technologies/ThermoFisher, cat #14040133) and blocked with 10% goat serum/PBS for 1 h. Subsequently, sections were incubated with a chicken anti-GFP polyclonal antibody (Aves Laboratories, Tigard, Oregon, USA cat #GFP-1020) (1:500) overnight at 4 °C followed by incubation with goat anti-chicken Alexa Fluor 594 (Invitrogen/ThermoFisher cat #A11042) (1:500) at room temperature for 2 h. All antibody solutions were made in 1% goat serum/0.3% Triton X-100 in PBS. After DPBS Ca/Mg washes, slides were mounted with DAPI-containing Fluoro-Gel II (Electron Microscopy Science, Hatfield, PA, USA cat #17985-50). No primary antibody controls were run in parallel. Images were captured on the IX71 Olympus microscope, DP80 monochrome camera and cellSens software described above.

### Statistical analysis

Averages values are expressed as mean ± SE. The significance of experimental changes was analyzed using Student’s *t*-test as unpaired data using the SigmaPlot 13.0 statistical software (Systat Software, San Jose, CA, USA). For the calculation of *p* values, all technical replicates from all biological replicates were used. The difference was considered statistically significant when the one-tailed *p*-value was <0.05.
